# Integrated Computational Solution for Predicting Skin Sensitization Potential of Molecules

**DOI:** 10.1371/journal.pone.0155419

**Published:** 2016-06-07

**Authors:** Konda Leela Sarath Kumar, Sujit R. Tangadpalliwar, Aarti Desai, Vivek K. Singh, Abhay Jere

**Affiliations:** 1 LABS, Persistent Systems Limited, Pune, Maharashtra, India; 2 Department of Pharmacoinformatics, National Institute of Pharmaceutical Education and Research (NIPER), Hajipur, Vaishali, Bihar, India; ENEA, ITALY

## Abstract

**Introduction:**

Skin sensitization forms a major toxicological endpoint for dermatology and cosmetic products. Recent ban on animal testing for cosmetics demands for alternative methods. We developed an integrated computational solution (SkinSense) that offers a robust solution and addresses the limitations of existing computational tools i.e. high false positive rate and/or limited coverage.

**Results:**

The key components of our solution include: QSAR models selected from a combinatorial set, similarity information and literature-derived sub-structure patterns of known skin protein reactive groups. Its prediction performance on a challenge set of molecules showed accuracy = 75.32%, CCR = 74.36%, sensitivity = 70.00% and specificity = 78.72%, which is better than several existing tools including VEGA (accuracy = 45.00% and CCR = 54.17% with ‘High’ reliability scoring), DEREK (accuracy = 72.73% and CCR = 71.44%) and TOPKAT (accuracy = 60.00% and CCR = 61.67%). Although, TIMES-SS showed higher predictive power (accuracy = 90.00% and CCR = 92.86%), the coverage was very low (only 10 out of 77 molecules were predicted reliably).

**Conclusions:**

Owing to improved prediction performance and coverage, our solution can serve as a useful expert system towards Integrated Approaches to Testing and Assessment for skin sensitization. It would be invaluable to cosmetic/ dermatology industry for pre-screening their molecules, and reducing time, cost and animal testing.

## Introduction

In cosmetic industry, one of the major determinant for topical products is ‘skin sensitization’[1]. Usually the term ‘skin sensitization’ refers to heightened immune response in susceptible individuals on topical exposure to a molecule[2]. Conventionally, Buehler guinea pig test (BGPT), guinea pig maximization test (GPMT) and more recently the murine local lymph node assay (LLNA)[3] are used to assess the skin sensitization potential of a molecule. However, animal testing for cosmetic ingredients is banned in European Union[4], and the REACH (Registration, Evaluation and Authorization of Chemicals) policy[5] enforces that companies assess, manage and communicate the risks associated with molecules manufactured by them. Considering these circumstances, there is an urgent need to devise alternative methods that can reduce the effort and cost, and more importantly, eliminate the usage of animals in cosmetic research. The recently published Adverse Outcome Pathway (AOP) for skin sensitization by OECD[6] summarizes the causal links between molecular initiaing event of skin sensitization (i.e. modification of skin protein by a molecule), intermediate key events and the adverse outcome at biological level[7]. This mechanistic knowledge offers opportunity to develop efficient methods or map existing ones (*in vitro*, *in chemico* or *in silico*) for assessing skin sensitization without the need for animal testing[7]. For e.g. The *in vitro* assays such as KeratinoSens^TM^[8,9] and human-Cell line Activation Test (h-Clat)[10] were mapped to particular key events of this AOP [11,12].

Computational (*in silico*) approach, due to its cost- and resource- efficiency, could be an alternative to *in vivo* and possibly *in vitro* evaluation of skin sensitization potential with reference to AOP[13,14]. This approach includes the use of statistical, mechanism based and knowledge based methodologies to predict the skin sensitization potential of molecules[15,16]. The ‘Statistical Approach’ uses: (1) already available skin sensitization data to select appropriate molecular descriptors (e.g. number of nitrogen atoms, number of double and triple bonds, etc.); and (2) regression or classification algorithms for classifying test molecules into sensitizers and non-sensitizers[17]. The ‘Mechanism Based’ approach utilizes heats of reaction[18], Taft coefficients or experimental measures of reactivity with nucleophiles to correlate with skin sensitization potential of molecules[17] while the ‘Knowledge Based’ approach usually uses rules (alerts) devised by domain experts. Usually an ‘alert’ is prediction of a toxicophore that could be potentially associated with skin sensitization, and is derived from chemical grouping or empirical rules[17].

The three approaches stated above are incorporated in (Quantitative) Structure Activity Relationship [(Q)SAR] models and expert systems designed to predict skin sensitization potential of molecules. Skin sensitization (Q)SAR model refers to a mathematical equation that relates chemical structure (or properties) of molecules to skin sensitization potential in a quantitative manner[19,20]. On the other hand, expert systems are encoded in the form of rules, used for evaluating skin sensitization potential. These rules are derived by using either expert judgment (e.g. DEREK), statistical inference (e.g. Case Ultra, TOPKAT and VEGA) or combination of both i.e. hybrids (e.g. TIMES-SS)[21].

A recent report evaluating[21] the performance of Case Ultra, TOPKAT, DEREK, VEGA v2.1.3, TIMES-SS v2.27, Toxtree and the OECD (Q)SAR toolbox v3.1 showed that these models suffer from: (1) unsatisfactory performance, i.e. high rate of false positives; and/or (2) limited coverage, i.e. only small sub-set of the test molecules were reliably predicted. Another study evaluating DEREK, TOPKAT and TOPS-MODE also reported similar findings i.e. high sensitivity but poor specificity[22]. We believe that limited dataset in terms of either size or diversity, and the lack of mechanistic knowledge in the prediction models could be major contributory factors for these limitations.

To address these limitations and offer a robust solution, we have developed a new approach for predicting skin sensitization potential of molecules. The novelty of our approach lies primarily in the incorporation of three important components for prediction: (1) multiple QSAR models, which were built using large publicly available data on sensitizers (of various potency classes) and non-sensitizers; (2) structural similarity to known sensitizers and non-sensitizers; and (3) presence of sub-structure(s) associated with skin sensitization reaction mechanisms. This allowed us to integrate two complementary approaches i.e. statistical and mechanistic by a unique strategy, which helped achieve improved prediction performance and coverage. We tested our predictions on a challenge set[21] and obtained prediction accuracy of 75.32%, Correct Classification Rate (CCR) = 74.36%, sensitivity = 70.00% and specificity = 78.72%. Our results are far better as compared to widely used tool VEGA v1.08, which showed an accuracy of only 44.12%. To summarize, our integrated skin sensitization prediction solution ‘SkinSense’ has improved accuracy, better sensitivity and more specificity compared to the currently available solutions.

## Methods

### Building of QSAR Models for Skin Sensitization

**Fig 1:**
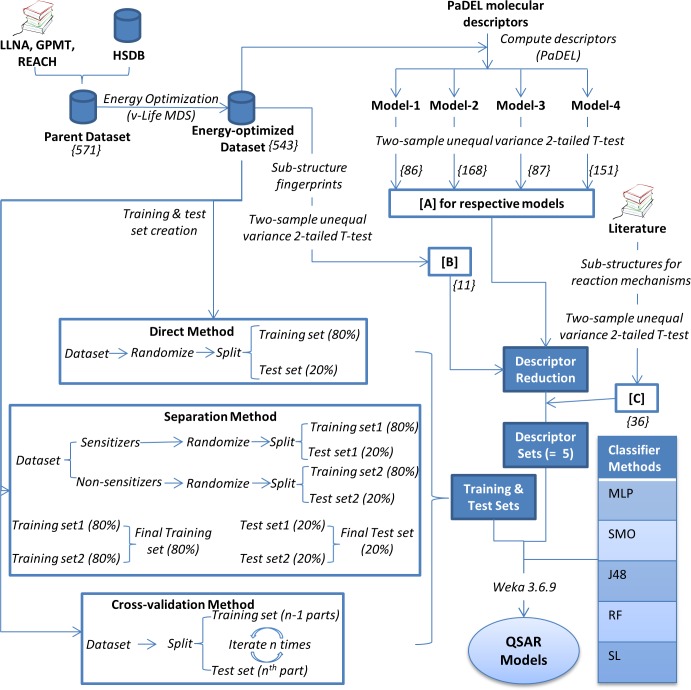
Steps followed for building QSAR models. QSAR: Quantitative Structure-Activity Relationship; GPMT: Guinea Pig Maximization Test; HSDB: Hazardous Substance DataBase; LLNA: Local Lymph Node Assay; REACH: Registration, Evaluation and Authorization of Chemicals; MLP: Multi-Layer Perceptron; RF: Random Forest; SL: Simple Logistic; SMO: Sequential Minimal Optimization; Numbers in curly brackets represent the count of respective entities (i.e. molecules, descriptors and fingerprints).

Briefly, building of QSAR models for skin sensitization involves: (1) collation of available skin sensitization data; (2) selection, computation and reduction (if required) of suitable descriptors (e.g. chemical, topological) and fingerprints; (3) creation of datasets for training and testing classifiers; and (4) applying appropriate classifier methods to differentiate sensitizers from non-sensitizers. Fig 1 and the description below elaborates the building of our QSAR models in accordance with best practices documented by Tropsha *et al*.[23].

#### Collation of skin sensitization dataset

Availability of data regarding molecules already characterized for skin sensitization potential is primary necessity for building, training and testing of QSAR model. This data was collated from literature and the Hazardous Substance DataBase (HSDB), which contains peer-reviewed compilation of toxicology data [24]. While collating data, only those molecules were considered which were tested for skin sensitization using LLNA and GPMT tests[25–29], and REACH allergens listed by Schubert (http://www.istas.ccoo.es/descargas/alergenos_REACH%5B1%5D%28230708%29.pdf). To ensure reliability, the data used for building models were curated using the following criteria: (1) whether molecule is tested by LLNA in addition to other suitable assays considering that LLNA is the preferred method; (2) whether classification of a molecule (i.e. sensitizer or non-sensitizer) supported by latest research article; and (3) whether CAS registry number for a molecule is unique. Based on this screening, we identified 571 unique molecules systematically characterized for their skin sensitization potential and denoted this as the ‘parent set’.

Molecules in the parent set were further classified (see Table 1) as Extreme (X), Strong (St), Moderate (M), Weak (W) and Non-sensitizer (N) as reported by Johansson *et al*. [29], Kern *et al*. [27],Cronin *et al*. [26], Gerberick *et al*. [25], Enoch *et al*.[28] and HSDB[24], whereas only as Sensitizers (S) and Non-sensitizers (N) by Schubert (http://www.istas.ccoo.es/descargas/alergenos_REACH%5B1%5D%28230708%29.pdf).

**Table 1:** Datasets used for building QSAR models.

	Parent set	E_o_^a^	Model-1	Model-2	Model-3	Model-4
X^b^	18	17	17	17		
St^c^	32	32	32	32		
S^d^	206	180		180		
M^e^	90	90			90	
W^f^	74	74				74
N^g^	151	150	49	150	150	150
*Total*	*571*	*543*	*98*	*379*	*240*	*224*

^a^Energy-optimized set

^b^Extreme

^c^Strong

^d^Sensitizer with unknown potency

^e^Moderate

^f^Weak

^g^Non-sensitizer.

Also, building an effective QSAR model requires complete spatial depiction of molecules^18^; thus, three dimensional (3D) structures for molecules in the parent set were determined. For this, SMILES (Simplified Molecular Input Line Entry System) representation of the molecules were converted to 2D SDF (2-Dimensional Structure Data File) using OpenBabel 2.3.2[30], followed by conversion to 3D MOL2 format and optimization of their energies using vLife-MDS[31] with Merck Molecular Force Field (MMFF)[32,33]. Structure of 28 out of 571 molecules could not be optimized using vLife-MDS[31], and hence, they were excluded from further analysis. The remaining 543 molecules were considered for building QSAR models, and henceforth, referred to as ‘Energy-Optimized set (E_o_)’.

Using E_o_, we built four separate QSAR models to ensure that the characteristics of molecules with different potency classes were captured appropriately. As shown in Table 1, model-1 was built with Extreme (X) and Strong (St) sensitizers, while model-3 with Moderate (M) and model-4 with Weak (W) sensitizers respectively. Model-2 represented a generalized model for X, St and S (i.e. sensitizers with unknown potency). For model-1, all the extreme (= 17) and strong (= 32) sensitizers, and 49 non-sensitizers from E_o_ with least average similarity were considered. All non-sensitizers were not considered in order to avoid biasing the model towards non-sensitizers[23]. For other three models, all the available sensitizers and all the non-sensitizers were considered. Weka 3.6.9[34] (henceforth referred to as Weka) was used for building these models.

#### Descriptor selection, computation and reduction

Descriptors are the properties (for e.g. chemical, topological or geometrical) that can characterize a molecule[35]. In a QSAR model, differences in the values of descriptors are used for differentiating sensitizers from non-sensitizers. As discussed below, we relied on statistical analysis to select descriptors whose values differ significantly between sensitizers and non-sensitizers.

PaDEL 2.15[36] is a free and open source software for calculating molecular descriptors and fingerprints. It offers a total of 863 molecular descriptors and 9365 fingerprints, and all were considered as starting set for building our QSAR models. Using T-test (two-sample unequal variance 2-tailed), molecular descriptors whose values differ significantly (i.e. 95% confidence interval) between sensitizers and non-sensitizers were selected for each QSAR model. This set of descriptors was called as [A] and had 86, 168, 87 and 151 descriptors for models-1, 2, 3 and 4 respectively.

Further, to incorporate literature-derived mechanistic details for skin sensitization in our QSAR models, we selected suitable sub-structure fingerprints[37] from PaDEL 2.15[36]. Eleven of them represented skin protein reactive groups, and differed significantly between sensitizers and non-sensitizers in 164 molecules of E_o_[28]. This set of sub-structure fingerprints is hereafter referred to as [B] and T-test, as elaborated above, was used to identify them. An additional 36 sub-structure fingerprints were selected from literature based on reports indicating their association with skin sensitization reaction mechanisms[38–40]. This set of sub-structure fingerprints is hereafter referred to as [C]. See Tables A-E in S1 File for the descriptors and fingerprints contained in [A], [B] and [C] for each QSAR model.

**Fig 2:**
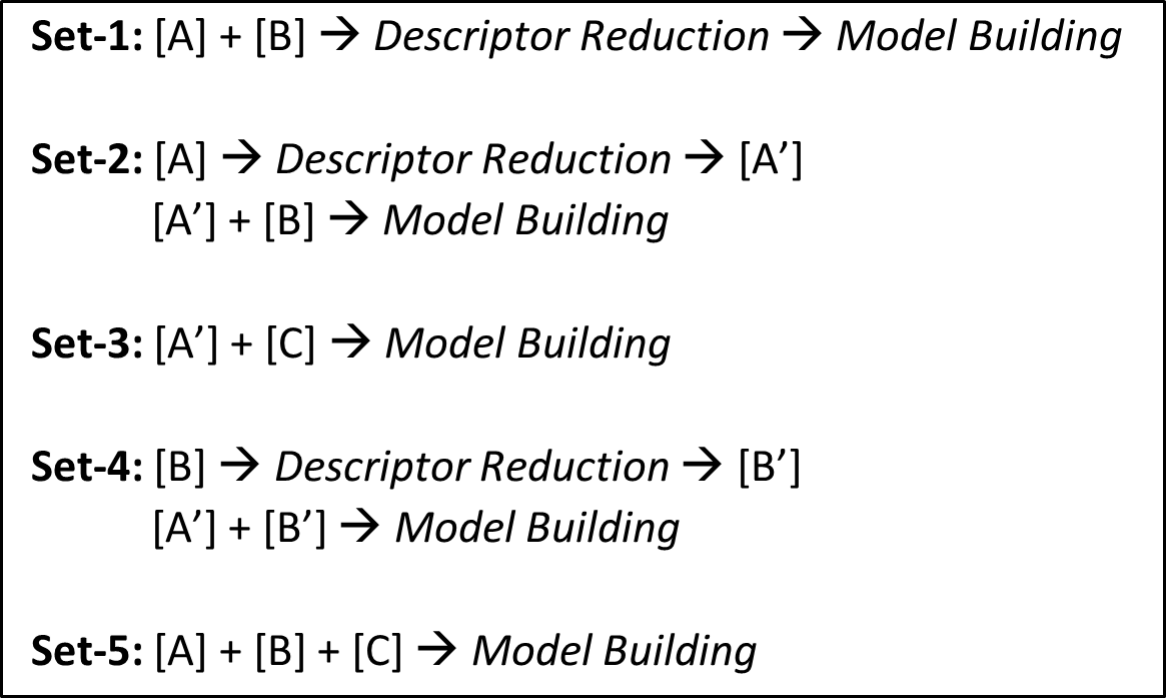
Descriptor sets used for QSAR models.

To further refine the sets of descriptors and fingerprints such that they are mutually independent (i.e. changing the value of one descriptor does not have an impact on another descriptor)[35], reduction protocol was performed using Weka[34]. *CfsSubsetEval* module along with BestFirst method was used for this analysis [41]. We defined 5 combinations (sets) of descriptors and fingerprints for descriptor reduction as depicted in Fig 2. Set-1 contains the descriptors from combination of [A] and [B], followed by subsequent reduction. This allowed selection of important yet independent descriptors and fingerprints from [A] and [B] respectively. Sets-2 and 3 ensured that all the fingerprints in [B] and [C] respectively were considered as they were derived from literature, and combined with the independent descriptors from [A] i.e. [A’]. Set-4 conserved all the independent descriptors i.e. [A’] and independent fingerprints from [B] i.e. [B’] respectively, and set-5 allowed all the selected descriptors and fingerprints for the building of QSAR model (i.e. [A] + [B] + [C]). It is important to note that descriptor reduction was not performed for [C] to ensure that all the literature-derived fingerprints associated with skin sensitization mechanisms were retained. Furthermore, for [A], descriptors pertaining to the corresponding models (i.e. 86, 168, 87 and 151 descriptors for models-1, 2, 3 and 4 respectively) were used.

#### Creation of training and test sets

Training and test sets are required for training the classifier methods to differentiate sensitizers from non-sensitizers, and for testing the performance of models. The molecules considered for building each model were divided into training and internal test sets by employing three methods: (1) direct method (D); (2) separation method (S); and (3) cross-validation method (C). Weka[34] was used for performing these computations.

Briefly, direct method involves randomizing the dataset, followed by splitting it into training and test set with 80% and 20% molecules in them respectively. However, direct method does not guarantee proportional distribution of sensitizers and non-sensitizers in training and test sets; hence, we devised ‘separation method’ to ensure proportional distribution. In separation method, sensitizers and non-sensitizers were pre-segregated manually in two separate files and were provided as input to Weka[34]. The randomization and splitting of these datasets into 80% and 20% molecules was done using Weka[34], and the resulting files were used to create training and test sets with 80% and 20% molecules in them respectively. Cross-validation method involves splitting of dataset into n (= 10 in our case) parts, out of which n-1 parts are used as training set and the n^th^ part is used as test set. This is iterated until all the parts are used as test set once (see Fig 1).

An additional test set, called ‘representative test set (RTS)’, was created from E_o_ by selecting equal number of sensitizers and non-sensitizers in following proportion: X = 10, St = 10, S = 10, M = 10, W = 10 and N = 50. The molecules in each class were chosen on the basis of mutually diverse chemical structures as deduced from Tanimoto coefficient[42].

To benchmark our QSAR models with routinely used tools, a new test set was created by collating molecules from literature, that were not part of dataset used earlier. We collated 76 unique molecules from literature[25–27,43–46], out of which 2 molecules failed at optimization step described earlier and hence were excluded from further study. The remaining 74 molecules were used as ‘challenge set-1’. This test set contained molecules in following proportion: X = 0, St = 10, S = 35, M = 2, W = 11 and N = 16.

Another dataset of 100 molecules, used by Teubner *et al*.[47] for comparing existing skin sensitization models, was also considered. This set comprised of 45 sensitizers of varying potency and 55 non-sensitizers. Out of these, 19 molecules were present in our parent set, and thus, were removed. Furthermore, 1 sensitizer (CAS number: 52408-42-1) failed during conversion to 2D SDF from SMILES using OpenBabel 2.3.2[30], and 2 sensitizers (CAS numbers: 1307-96-6 and 7758-89-6) and a non-sensitizer (CAS number: 30989-05-0) failed to optimize using vLife-MDS[31]. The remaining 77 molecules i.e. 30 sensitizers and 47 non-sensitizers constituted ‘challenge set-2’.

#### Classifier methods for QSAR models

Classifier methods are required for QSAR models to help classify test molecules into sensitizers and non-sensitizers. Following classifier methods from Weka[34] were chosen[48–52]: MLP (Multi-Layer Perceptron)[48], SMO (Sequential Minimal Optimization)[49], J48[50], RF (Random Forest)[51] and SL (Simple Logistic)[52]. MLP is a representative of artificial neural network, SMO for support vector machine, J48 for decision tree, RF for ensemble of decision trees and SL for logistic regression. All classifier methods were used with default parameters except for RF where 100 trees (i.e. I = 100) was used based on earlier recommendation[53].

### Consensus Prediction Using QSAR Models, Similarity Information and Sub-Structure Pattern

**Fig 3:**
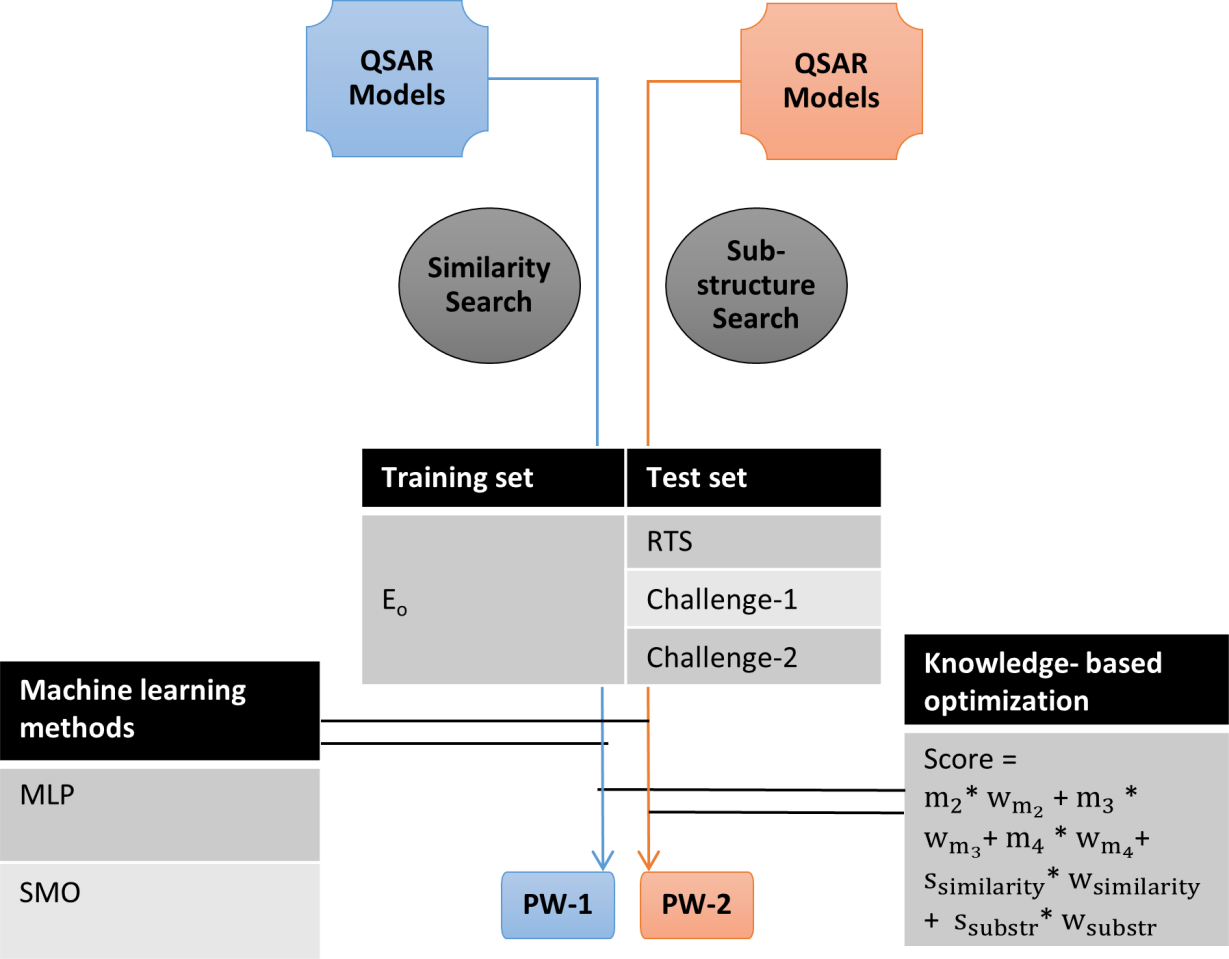
Integration of QSAR models, similarity information and sub-structure pattern into prediction workflows (PWs). Blue and red colors depict components that differ in the two Prediction Workflows, PW-1 and PW-2. Components in black and grey are those that are common in both PW-1 and PW-2. QSAR: Quantitative Structure-Activity Relationship; MLP: Multi-Layer Perceptron; SMO: Sequential Minimal Optimization; E_o_: Energy-optimized dataset; RTS: Representative test set; Challenge-1: Challenge set-1; Challenge-2: Challenge set-2; m_2_, m_3_, m_4_, s_similarity_ and s_substr_ are predictions from QSAR. models-2, 3 and 4, similarity information and sub-structure pattern, and w_m2_, w_m3_, w_m4_, w_similarity_ and w_substr_ are their corresponding weights.

A unique aspect of our study is the integration of QSAR models (see section titled “Integrated prediction workflows” for detail on selection of models), similarity information (see section titled “Identification of structurally similar molecules in the dataset” for detail) and sub-structure pattern (see section titled “Identification of sub-structures associated with skin sensitization reaction mechanisms” for detail) into ‘Prediction Workflows’ (PW) for classifying a molecule as sensitizer or non-sensitizer. To achieve this integration, we employed two approaches: machine learning methods available in Weka[34] and knowledge-based (KB) optimization[54,55] (see Fig 3).

Amongst machine learning methods, MLP and SMO (i.e. implementation of support vector machine) were used due to their suitability for the optimization of QSAR models[56]. E_o_ was used for training using cross-validation method (with n = 10 in our case) (see Table F in S1 File for details). The performance of these classifiers were evaluated using E_o_ and RTS, and challenge sets-1 and 2 in Weka[34].

For knowledge-based optimization[54,55], a weighted sum of predictions from components i.e. QSAR models, similarity information and sub-structure pattern was used to compute the final score of a test molecule (see Fig 3 and Eq 1). In this, scores from prediction components (e.g. QSAR models m_2_, m_3_ and m_4_) were multiplied by their corresponding weights (e.g. w_m2_, w_m3_, w_m4_). The weights refer to relative performance of the QSAR models and importance of similarity information and sub-structure pattern in determining skin sensitization potential (see Table 2). In Eq 1, m_2_, m_3_ and m_4_ are predictions from QSAR models-2, 3 and 4 (see sections titled “Evaluation of QSAR model variants” and “Integrated prediction workflows” for details on selection of QSAR models), and s_similarity_ and s_substr_ are those from similarity information and sub-structure pattern respectively. w_m2_, w_m3_, w_m4_, w_similarity_ and w_substr_ are their corresponding weights. The prediction from QSAR models (i.e. m_2_, m_3_ and m_4_) was scored as 1 (for test molecule predicted as sensitizer) or -1 (for test molecule predicted as non-sensitizer). Prediction scores of similarity information and sub-structure pattern are described in sections titled “Identification of structurally similar molecules in the dataset” and “Identification of sub-structures associated with skin sensitization reaction mechanisms” respectively. If the resultant sum was positive, molecule was predicted as a sensitizer; if negative, the molecule was predicted as non-sensitizer; and if zero, the prediction was indeterminate. Table G in S1 File summarizes the weights used and the corresponding prediction performance.

**Table 2:** Weights used for components of prediction workflows in knowledge-based optimization.

	QSAR Model-2		QSAR Model-3		QSAR Model-4		Similarity Information				Sub-structure Pattern	
	S^a^	N^b^	S	N	S	N	S	N	NS^c^	No Match^d^	Present^e^	Absent^f^
KB-a	1	1	0.4	0.4	0.3	0.3	1	1	0	0	1	0.5
KB-b	1	0.8	0.8	0.4	0.3	0.4	1	1	0	0	1	0.5
KB-c	1	0.8	0.6	0.4	0.3	0.4	1	1	0	0	1	0.5
KB-d	1	0.8	0.4	0.3	0.3	0.3	1	1	0	0	1	0.5

^a^Weight used if test molecule is predicted to be a ‘sensitizer’

^b^Weight used if test molecule is predicted to be a ‘non-sensitizer’

^c^Weight used if test molecule shows equal similarity to both, sensitizer and non-sensitizer

^d^Weight used if test molecule is not similar to any molecule in our parent set

^e^Weight used if sub-structure is found in a test molecule

^f^Weight used if sub-structure is not found in a test molecule.

### Identification of Structurally Similar Molecules in the Dataset

As a component of our prediction workflows, test molecules were screened for their structural similarity (i.e. ‘similarity information’) to known sensitizers and non-sensitizers contained in parent set. Similarity was estimated by computing Tanimoto coefficient[42] using Pybel with path-based fingerprint (FP2)[57] approach. Based on earlier recommendations, Tanimoto coefficient cutoff was set to 0.6 (i.e. 60% similarity)[58,59]. Test molecules failing this cutoff were scored as 0, while the molecules passing this cutoff (i.e. with coefficient ≥ 0.6) were scored as 1 or -1 depending on whether they show highest similarity to sensitizer(s) or non-sensitizer(s) respectively. Molecules showing equal similarity (i.e. equal Tanimoto coefficient value) to both, sensitizers and non-sensitizers, were also scored as 0.

Moreover, for molecules appearing identical to any dataset molecule (i.e. 100% similarity) based on Tanimoto coefficient, their InChIKey[60] values were compared by pattern matching to ascertain that the molecules were indeed identical.

### Identification of Sub-Structures Associated with Skin Sensitization Reaction Mechanisms

As our objective was to devise an integrated workflow to predict skin sensitization potential of molecules, we included ‘sub-structure pattern’ as a key component to identify chemical groups known to react with skin proteins[28] i.e. associated with skin sensitization reaction mechanisms. For this, SMILES of test molecules were compared against set of SMARTS (SMiles ARbitrary Target Specification) patterns collated from literature[28] using Pybel[57]. If a test molecule contained any such group i.e. matched with any SMARTS pattern then it was scored as 1, else -1. Absence of a sub-structure was scored as -1 and not 0 because it is an indicative that the molecule may not interact with skin proteins, and hence, would not be a sensitizer.

### Computation of Performance Measures

Prediction performance of the models and prediction workflows were gauged by following measures: accuracy, sensitivity, specificity and CCR[48,61,62]. Sensitivity was computed as proportion of correctly predicted sensitizers (see Eq 2), and specificity as proportion of correctly predicted non-sensitizers (see Eq 3). Accuracy was computed as ratio of correctly predicted molecules (both sensitizers and non-sensitizers) as compared to all molecules included in the analysis (see Eq 4). CCR was computed as average of the rates correctly predicted within each class (see Eq 5). In Eqs 2, 3 and 4, True Positive (TP) was described as number of sensitizers correctly predicted as sensitizers, False Positive (FP) as number of non-sensitizers wrongly predicted as sensitizers, True Negative (TN) as number of non-sensitizers correctly predicted as non-sensitizers and False Negative (FN) as number of sensitizers wrongly predicted as non-sensitizers.

$$\begin{array}{c} If\\ Score>0,\;Prediction\;=\;Sensitizer\\ Score\;<\;0,\;Prediction\;=\;Non\;-\;sensitizer\\ Score\;=\;0,\;Prediction\;=\;Indeterminate\\ where\\ Score\;=\;m_{2}*w_{m}{}_{{}_{2}}+\;m_{3}*\;w_{m}{}_{{}_{3}}+m_{4}*w_{m_{4}}\\ +s_{similarity}*w_{similarity}+s_{substr}*w_{substr} \end{array}$$ Equation 1

$$Sensitivity\;=\;\frac{TP}{TP+FN}$$ Equation 2

$$Specificity\;=\;\frac{TN}{TN+FP}$$ Equation 3

$$Accuracy\;=\;\frac{TP+TN}{TP+FN+TN+FP}$$ Equation 4

$$\mathrm{CCR}\;=\;\frac{\mathrm{Specificity}+\mathrm{Sensitivity}}{2}$$ Equation 5

## Results and Discussion

### Quantitative Structure-Activity Relationship (QSAR) Models

As stated earlier, we built four QSAR models. For each model, a total of 75 variants (= 1 model x 5 descriptor sets x 3 methods of separating training and test sets x 5 classifier methods) were built using various combinations of descriptor and fingerprint sets, segregation of training and test sets, and classifier methods (see Methods for details). This resulted in a total of 300 variants (= 4 models x 75 variants for each model); thus, allowing us to evaluate combinatorial list of QSAR models and choose the best performing ones.

Nomenclature for each variant (e.g. 1D4RF) was represented by an alphanumeric code as elaborated below: <model i.e. [1–4]><method used for segregating training and test sets i.e. [D|S|C]><descriptor set used i.e. [1–5]><classifier method i.e. [J48|MLP|RF|SL|SMO]>. In 1D4RF, ‘1’ refers to QSAR model-1, ‘D’ is direct method for segregating training and test sets, descriptor set-‘4’ for building and random forests ‘RF’ as the classifier method.

### Evaluation of QSAR Model Variants

All the 300 variants were evaluated for prediction performance using their respective test sets and RTS. The respective test sets of each variant contains sensitizers of specific categories (for e.g. extreme (X) and strong (St) for variants of model-1) and non-sensitizers (see sections titled “Collation of skin sensitization dataset” and “Creation of training and test sets” for details). On the other hand, RTS contains equal number of sensitizers of all categories (i.e. extreme (X), strong (St), moderate (M), weak (W) and sensitizers of unknown potency (S)) and equal number of non-sensitizers (see 2.1.3 for details). Thus, this analysis revealed the ability of variants to predict sensitizers in their respective categories as well as sensitizers across all the categories. For instance, among the variants of model-1, 1D1RF, 1D4RF and 1D3J48 showed highest prediction accuracy (= 100%) for their respective test sets. However, with respect to RTS, 1C1RF and 1C4RF showed highest accuracy (= 84%) while 1D1RF, 1D4RF and 1D3J48 showed accuracies of 81%, 81% and 77% respectively (see Table H in S1 File).

**Fig 4:**
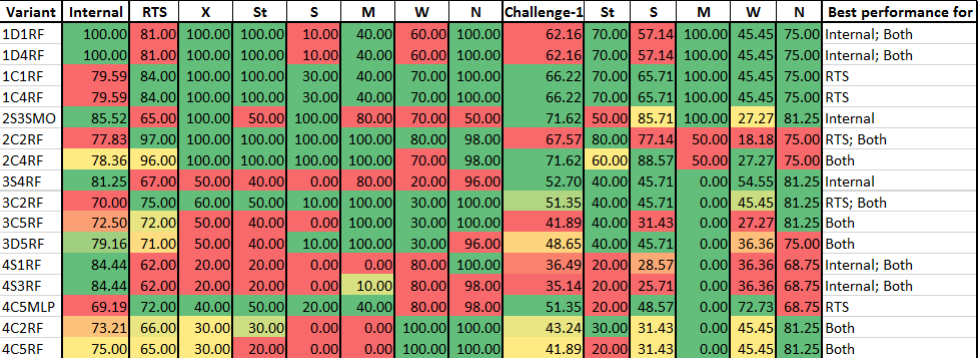
Percent prediction accuracy of short-listed variants of models. Color-coded scale from green to red indicates decreasing prediction accuracy. RTS and Challenge-1 sets are expanded to show the prediction accuracy for each category of sensitizers and non-sensitizers. Internal: Internal test set; RTS: Representative test set; Challenge-1: Challenge set-1; Both: Internal & RTS; X: Extreme; St: Strong; S: Sensitizer with unknown potency; M: Moderate; W: Weak; N: Non-sensitizer.

Overall, prediction accuracies of the variants ranged between 57.77%-100% with respect to their respective test sets (variants 4D4J48, and 1D1RF, 1D3J48 and 1D4RF respectively) and 44%-97% for RTS (variants 3C2SL, and 2C1RF and 2C2RF respectively) (see Table H in S1 File). For further evaluation, we short-listed the variants showing best accuracy with respect to: (1) internal test set; (2) RTS; and (3) a combination of internal test set and RTS. Fig 4 shows the variants that satisfy above criteria.

To assess the robustness of variants, we evaluated their performance on ‘new set of molecules’ (i.e. molecules not used during model building). Challenge set-1 containing 74 molecules collated from literature (see 2.1.3 for details) was used for this evaluation. As shown in Fig 4, 2S3SMO and 2C4RF showed the highest prediction accuracy (= 71.62%) followed by that of 2C2RF (= 67.57%). This implied that variants of model-2 performed best on an external test set, which was expected owing to the diversity of molecules used for building them.

On comparing the prediction accuracy for individual categories of sensitizers from RTS and challenge set-1, it was evident that a single model cannot predict all the types of sensitizers with equal accuracy. In particular, variants of models-1 and 2 could predict X, St and M better than W, while variants of models-3 and 4 were better at predicting M and W respectively. Thus, to enhance the spectrum of prediction and improve the overall performance, we integrated individual models into prediction workflows as elaborated below.

### Integrated Prediction Workflows

As stated above, we built prediction workflows by combining best performing variants from each QSAR model such that the overall prediction performance improves. Criteria used for selecting these variants were as follows: (1) prediction performance on their respective test sets and RTS; (2) ability to predict sensitizer categories used to train the variants; and (3) ability to predict other sensitizer categories. For e.g. 3C2RF was preferred over 3C5RF owing to better prediction of other sensitizer categories (i.e. X and St), though, they predicted their respective sensitizer category (i.e. M) with equal accuracy. Moreover, no variant of model-1 was used in prediction workflow as variants of model-2 compensated for it by predicting X and St with equal or better accuracy (see Fig 4).

Based on above criteria, the variants 2C2RF and 2C4RF of model-2, 3C2RF of model-3 and 4C2RF of model-4 were selected. These were combined such that each combination had variants from all the three QSAR models viz. (1) 2C2RF, 3C2RF and 4C2RF; and (2) 2C4RF, 3C2RF and 4C2RF. Y-randomization for these QSAR model variants were performed to assess robustness and eliminate chance correlation. For each variant, 10 randomization runs were performed as indicated by Garg *et al*.[63]. In all cases, the QSAR model variants based on real data showed much higher accuracy than the random models, indicating no chance correlation in our model variants (see Table I in S1 File).

In addition to the predictions from QSAR models, we incorporated following two factors. (1) ‘Similarity information’ of known sensitizers and non-sensitizers from our parent set based on the principle of similar-property, considering that structurally similar molecules exhibit similar properties[64]. (2) Identification of ‘sub-structure patterns’ associated with skin sensitization reaction mechanisms. This was done to identify the presence of chemical groups known to react with skin proteins[28,61].

#### Integration of predictions from components

We built two prediction workflows corresponding to following model variants: (1) 2C2RF, 3C2RF and 4C2RF; and (2) 2C4RF, 3C2RF and 4C2RF respectively. The predictions from each component of the workflow i.e. QSAR models, similarity information and sub-structure pattern were consolidated using machine learning methods (i.e. MLP and SMO) and knowledge-based optimization, with E_o_ as the training set. The details of score computation from each component and their integration is discussed in section titled “Consensus prediction using QSAR models, similarity information and sub-structure pattern”. It should be noted that weight of any sub-structure pattern was kept as 1 (when it is found in a test molecule) and 0.5 (when it is absent in a test molecule) to account for the fact that presence of a sub-structure is a good indicator of sensitizer while its absence does not necessarily imply that a test molecule is a non-sensitizer (see Table 2). As mentioned above, similarity information and sub-structure pattern were components of both of our prediction workflows.

### Evaluation of Prediction Workflows

Since, various methods were used to integrate the predictions in our workflows, their performance on E_o_, RTS dataset and challenge sets-1 and 2 were evaluated. The results are discussed in following sections:

#### Comparative performance of prediction workflows

Table 3 details the prediction performance of our prediction workflows (PW-1 and PW-2) with reference to the use of machine learning methods (MLP and SMO) and knowledge-based optimization. In this table, KB-a, KB-b, KB-c and KB-d refer to various combinations of weights assigned to scores from components (QSAR models, similarity information and sub-structure pattern) of our workflows (see Eq 1 and Table 2).

**Table 3:** Performance of prediction workflows with machine learning methods and knowledge-based optimization.

	Method	% Accuracy			
		E_o_^a^	RTS^b^	Challenge set-1	Challenge set-2
PW-1^c^	MLP^e^	100.00	100.00	70.27	67.53
PW-1 ^c^	SMO^f^	100.00	100.00	72.97	67.53
PW-1 ^c^	KB-a^g^	98.16	99.00	78.38	74.03
PW-1 ^c^	KB-b^g^	99.08	99.00	78.38	75.32
PW-1 ^c^	KB-c^g^	98.34	99.00	78.38	74.03
PW-1 ^c^	KB-d^g^	98.34	99.00	79.73	74.03
PW-2^d^	MLP	100.00	100.00	70.27	67.53
PW-2 ^d^	SMO	100.00	100.00	71.62	67.53
PW-2 ^d^	KB-a^g^	98.34	100.00	78.38	72.70
PW-2 ^d^	KB-b^g^	99.26	100.00	77.03	75.32
PW-2 ^d^	KB-c^g^	98.53	100.00	78.38	72.73
PW-2 ^d^	KB-d^g^	98.53	100.00	79.73	72.73

^a^Energy-optimized set

^b^Representative test set

^c^Prediction workflow containing following variants of models: 2C2RF, 3C2RF and 4C2RF

^d^Prediction workflow containing following variants of models: 2C4RF, 3C2RF and 4C2RF

^e^Multi-Layer Perceptron

^f^Sequential Minimal Optimization

^g^Knowledge-based optimization with different weights used for components of prediction workflows as elaborated in Table 2

As is evident from Table 3, MLP and SMO performed better than knowledge-based optimization on E_o_ and RTS for both the prediction workflows; however, the latter outperformed MLP and SMO on challenge sets-1 and 2. This may indicate over-fitting of the machine learning methods (i.e. MLP and SMO) to the training set, which is an inherent limitation of such methods. On the other hand, the better performance of knowledge-based optimization could be attributed to small set of parameters (i.e. 5) to be optimized and *a priori* understanding of their contributions to the skin sensitization potential of molecules. Among the knowledge-based (KB) optimized weights, KB-b weights showed the best performance.

With KB-b weights, PW-2 performed best with respect to E_o_ (accuracy = 99.26%) and RTS (accuracy = 100%). The prediction accuracy of PW-1 was also comparable i.e. 99.08% and 99% for respective test sets. For challenge set-1, PW-1 performed slightly better than PW-2 (accuracy = 78.38% and 77.03% respectively), while both the prediction workflows performed equally well with respect to challenge set-2 (accuracy = 75.32%).

Clearly, integration of QSAR models, similarity information and sub-structure pattern in prediction workflows performed better than individual QSAR models because even the best performing model showed lower accuracy on E_o_ and RTS than our prediction workflows (see Fig 4 and Table H in S1 File).

#### Assessment of prediction performance on challenge sets

Out of 74 molecules in challenge set-1, 58 (78.38%) and 57 (77.03%) were accurately predicted by PW-1 and PW-2 respectively. Among these, PW-1 accurately predicted 47 sensitizers while PW-2 predicted 46 sensitizers. Moreover, 11 non-sensitizers were accurately predicted by both the workflows. It is important to note that integration of QSAR models, similarity information and sub-structure pattern facilitated their correct classification by overcoming the incorrect predictions of individual computations. For e.g. in case of PW-1, 18 out of 47 correctly predicted sensitizers did not show similarity to any molecule in parent set. However, PW-1 predicted them correctly as sensitizers owing to identification of reactive group(s) and/or predictions from QSAR models. Similar trend was also observed for PW-2 (see Tables J and K in S1 File for details). Table L in S1 File lists the reactive groups predicted to be present on molecules of challenge sets-1 and 2 by our workflows.

PW-1 and PW-2 incorrectly predicted 16 (21.62%) and 17 (23.97%) molecules, of which 11 and 12 are sensitizers and the remaining 5 non-sensitizers. Among the sensitizers wrongly predicted as non-sensitizers by PWs, 4 contain reactive group(s), but 3 of them also showed similarity to non-sensitizers. Thus, consensus prediction by the workflow went wrong. Similarly, among the non-sensitizers wrongly predicted as sensitizers, reactive groups were absent in 4 molecules, but they showed similarity to sensitizers.

These results indicate that although the prediction workflows were able to overcome incorrect predictions by individual models in majority of the cases, there is a scope for further optimization of the relative contribution of individual predictions. Evaluation of challenge set-2 also led to similar conclusions (see Tables J and K in S1 File for details).

#### Contributions of QSAR models, similarity information and sub-structure patterns to prediction

From the results, it was evident that the predictions from QSAR models, similarity information and sub-structure pattern do not concur with each other in some cases. Thus, it was important to understand how these components contribute to overall prediction. For this, we performed ‘leave-one-out’ analysis for both the prediction workflows, wherein the QSAR models corresponding to PW-1 and PW-2 were considered in one category (A), similarity information in category B and sub-structure pattern in category C. Categories A, B and C were used in all possible combinations (i.e. A, B, C; A, B; A, C; and B, C) with E_o_, RTS and challenge sets-1 and 2 for this analysis.

As shown in Table 4, the performance on E_o_ and RTS decreased the most when similarity information was left out. On the other hand, performance on the challenge sets decreased the most when sub-structure pattern was removed. Similarity information appeared to be important for the internal sets (i.e. E_o_ and RTS) because the tested molecules were already present in the dataset, and thus, similar molecules could be found. This led to better performance when similarity information was included in the prediction workflow. Sub-structure pattern appeared to be an important contributor in correctly classifying the molecules of challenge sets, indicating that the chemical groups with potential to bind to skin proteins are an important determinant of skin sensitization potential of a molecule. It was also clear that removing QSAR models (i.e. category A) led to slight increase in the prediction accuracy for E_o_ (= 0.54% for PW-2) and RTS (= 1% for PW-1), but, it also led to indeterminate results for several molecules in all the test sets. Thus, QSAR models are key players in the prediction workflow.

**Table 4:** Leave-one out analysis to assess the contributions of QSAR models, similarity information and sub-structure pattern to the prediction performance of prediction workflows.

Dataset	Performance	A, B, C^a^		A, B^a^		A, C^a^		B, C^a^	
		PW-1^b^	PW-2^c^	PW-1^b^	PW-2^c^	PW-1^b^	PW-2^c^	PW-1^b^	PW-2^c^
E_o_^d^	Accuracy	99.08	99.26	99.82	99.82	91.53	91.16	99.80	99.80
	Sensitivity	98.73	98.98	99.75	99.75	88.30	87.79	100.00	100.00
	Specificity	100.00	100.00	100.00	100.00	100.00	100.00	99.02	99.02
	Indeterminate results^f^	0	0	0	0	0	0	48	48
RTS^e^	Accuracy	99.00	100.00	100.00	100.00	90.00	89.00	100.00	100.00
	Sensitivity	98.00	100.00	100.00	100.00	80.00	78.00	100.00	100.00
	Specificity	100.00	100.00	100.00	100.00	100.00	100.00	100.00	100.00
	Indeterminate results^f^	0	0	0	0	0	0	22	22
Challenge set-1	Accuracy	78.38	77.03	67.57	68.92	78.38	79.73	74.24	74.24
	Sensitivity	81.03	79.31	67.24	68.97	75.86	77.59	76.92	76.92
	Specificity	68.75	68.75	68.75	68.75	87.50	87.50	64.29	64.29
	Indeterminate results^f^	0	0	0	0	0	0	8	8
Challenge set-2	Accuracy	75.32	75.32	55.84	57.14	75.32	74.03	63.77	63.77
	Sensitivity	70.00	70.00	66.67	66.67	70.00	66.67	65.38	65.38
	Specificity	78.72	78.72	48.94	51.06	78.72	78.72	62.79	62.79
	Indeterminate results^f^	0	0	0	0	0	0	8	8

^a^A: QSAR models (i.e. 2C2RF, 3C2RF and 4C2RF for PW-1 and 2C4RF, 3C2RF and 4C2RF for PW-2); B: Similarity information; C: Sub-structure pattern

^b^Prediction Workflow-1

^c^Prediction Workflow-2

^d^Energy-optimized set

^e^Representative test set

^f^Prediction workflows could not classify the test molecule as sensitizer or non-sensitizer (i.e. score = 0).

Furthermore, it was interesting to note that leaving out similarity information led to an increase (= 2.7% for PW-2) in the accuracy for challenge set-1, though there was a decrease (= 1.3% for PW-2) in accuracy for challenge set-2. Thus, leave-one out analysis highlighted the importance of each component of the prediction workflows and indicated the scope for further improvement either in the thresholds used to differentiate sensitizers from non-sensitizers, the weights, or both of them. For example, a molecule from our dataset was considered similar to a test molecule if Tanimoto coefficient ≥ 0.6. Increasing this threshold would make the criterion more stringent, and may improve its contribution towards final prediction.

### Comparative Performance of PW-1 & 2 with Existing Tools

**Fig 5:**
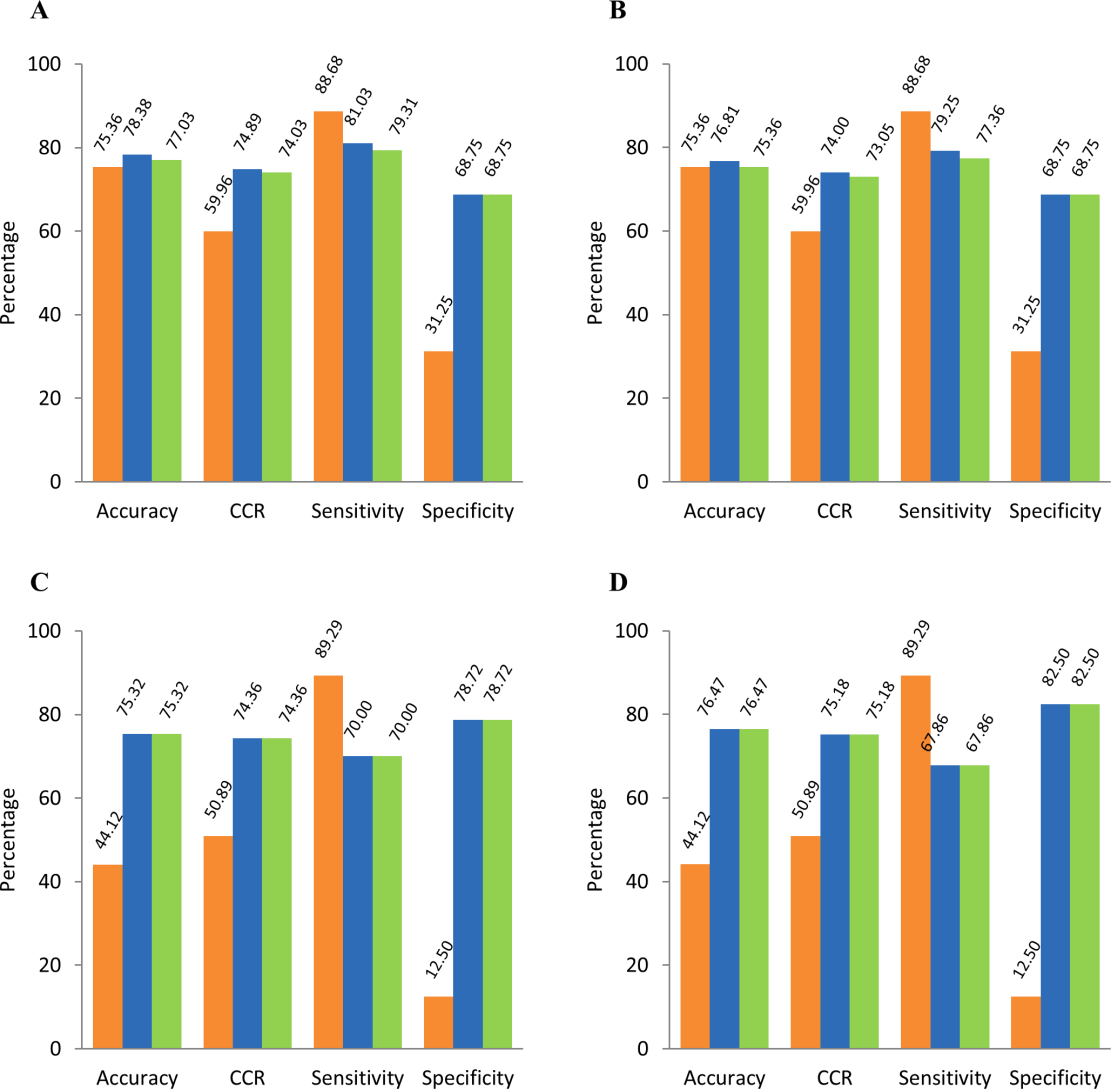
Comparative performance of our prediction workflows with VEGA v1.08. Panel A: Molecules of challenge set-1 processed by our prediction workflows (= 74) and VEGA v1.08 (= 69) used for computation; Panel B: 69 molecules of challenge set-1 processed by our prediction workflows as well as VEGA v1.08 were used for computation; Panel C: Molecules of challenge set-2 processed by our prediction workflows (= 77) and VEGA v1.08 (= 68) used for computation; Panel D: 68 molecules of challenge set-2 processed by our prediction workflows as well as VEGA v1.08 were used for computation. VEGA v1.08: orange bars; PW-1: blue bars; PW-2: green bars. CCR: Correct Classification Rate.

We evaluated the molecules of challenge sets using freely available tool, VEGA v1.08 (http://www.vega-qsar.eu/index.php). With respect to challenge set-1 containing 74 molecules, VEGA v1.08 could process 69 molecules, while PW-1 and 2 could process all the molecules. On comparing their prediction accuracies, our workflows showed slightly better performance (i.e. 3.02% by PW-1 and 1.67% by PW-2) as compared to VEGA v1.08 (see Fig 5A). Considering only the 69 molecules processed by VEGA v1.08, the prediction accuracies by both, VEGA v1.08 and PW-2 were equal (= 75.36%) and PW-1 showed slightly higher accuracy (= 76.81%) (see Fig 5B). However, comparing sensitivity and specificity indicated that VEGA v1.08 has high sensitivity (= 88.68%), but poor specificity (= 31.25%) as compared to that of our prediction workflows (sensitivity = 79.25% and 77.36% for PW-1 and PW-2 respectively and specificity = 68.75% for both PW-1 and PW-2).

With respect to challenge set-2 containing 77 molecules, VEGA v1.08 could process 68 molecules, while PW-1 and 2 processed all the molecules. Considering these 68 molecules, the prediction accuracies of our prediction workflows were equal and 32.35% higher than that of VEGA v1.08 (see Fig 5C and 5D). The trends for sensitivity and specificity were similar to that observed with challenge set-1. Sensitivity of VEGA v1.08 was high (= 89.29%), but specificity was very low (= 12.50%) as compared to that of our prediction workflows (sensitivity = 67.86% and specificity = 82.50% for both PW-1 and 2).

We also performed predictions using VEGA v1.08 with ‘High (AD Index > = 0.8)’ and ‘High and Medium (AD Index > = 0.6)’ for both, challenge sets-1 and 2. As shown in Tables M and N in S1 File, VEGA v1.08 showed lower prediction performance than our prediction workflows even with the use of ‘High’ (accuracy = 72% and CCR = 62.87%) or ‘High and Medium’ (accuracy = 70.27% and CCR = 58.50%) reliability scores for challenge set-1. The observation was similar for challenge set-2 as well (see Tables O and P in S1 File).

Furthermore, we compared the prediction performance of our workflows with other existing tools, namely, Case Ultra, TOPKAT, DEREK, TIMES-SS v2.27 and OECD (Q)SAR toolbox, by deriving their prediction performance from Teubner *et al*.[47] with respect to the molecules of challenge set-2 (see Table Q in S1 File for detailed derivation of prediction performance and results). For OECD (Q)SAR toolbox, we assumed presence of alert in a test molecule as sensitizer and its absence as non-sensitizer because there is no direct way of mapping the presence or absence of alerts to whether a molecule is sensitizer or non-sensitizer[47]. Our prediction workflows appeared to have best prediction performance (accuracy = 75.32% and CCR = 74.36% for both PW-1 and 2) among the compared tools followed by that of DEREK (accuracy = 71.05% and CCR = 69.13%) and TIMES-SS (accuracy = 69.33% and CCR = 67.78%). The prediction performance of OECD (Q)SAR toolbox were as follows: OASIS alert (accuracy = 65.79% and CCR = 60.45%) and OECD alert (accuracy = 61.84% and CCR = 55.94%). Our prediction workflows also showed highest sensitivity (= 70.00%), and specificity (= 78.72%) followed closely by DEREK (= 78.26%). A recently published QSAR model for skin sensitization reported CCR of 71–88% in differentiating sensitizers from non-sensitizers on separate external sets[65]. It would be interesting to evaluate this model against the test set used by Teubner *et al*.[47] for evaluating the performance of existing tools.

On comparing prediction performance of existing tools within their applicability domains, DEREK (accuracy = 72.73% and CCR = 71.44%) and TOPKAT (accuracy = 60% and CCR = 61.67%) still showed lower prediction performance than our prediction workflows (accuracy = 75.32% and CCR = 74.36%) (see Table Q in S1 File for details). Although, TIMES-SS showed better prediction performance (accuracy = 90% and CCR = 92.86%) than our workflows, only 10 out of the total 77 molecules (13%) of challenge set-2 could be processed by it, thus, indicating poor coverage. For Case Ultra, as shown by Teubner *et al*.[47], even with prediction performance in the applicability domain (i.e. known fragments), it could process only 20 sensitizers and 28 non-sensitizers out of the 100 molecule dataset[47], and correctly identified 60% of sensitizers and 71% of non-sensitizers. For OECD (Q)SAR toolbox, as stated by Teubner *et al*.[47], applicability domain is not applicable.

In summary, our prediction workflows showed improved prediction performance as compared to other existing tools. As is evident, following factors contributed to the better performance of our prediction workflows by overcoming the limitations of existing tools: (1) use of larger dataset with molecules from different skin sensitization potency classes to build separate QSAR models, which helped increase coverage of our prediction workflows; (2) incorporation of literature-derived mechanistic knowledge (in the form of. similarity information and sub-structure patterns) helped increase the specificity; and (3) combining the QSAR models with mechanistic knowledge in a weighted fashion improved the overall prediction accuracy.

### SkinSense: Implementation of Prediction Workflow as Software

Owing to improved accuracy of our prediction workflows, we believe that they may fit in the role of expert system as a part of Integrated Approaches to Testing and Assessment (IATA) for skin sensitization[66]. Towards this, we have implemented our prediction workflow (PW-2 in particular) in a software application called SkinSense, using Java Swing technology.

**Fig 6:**
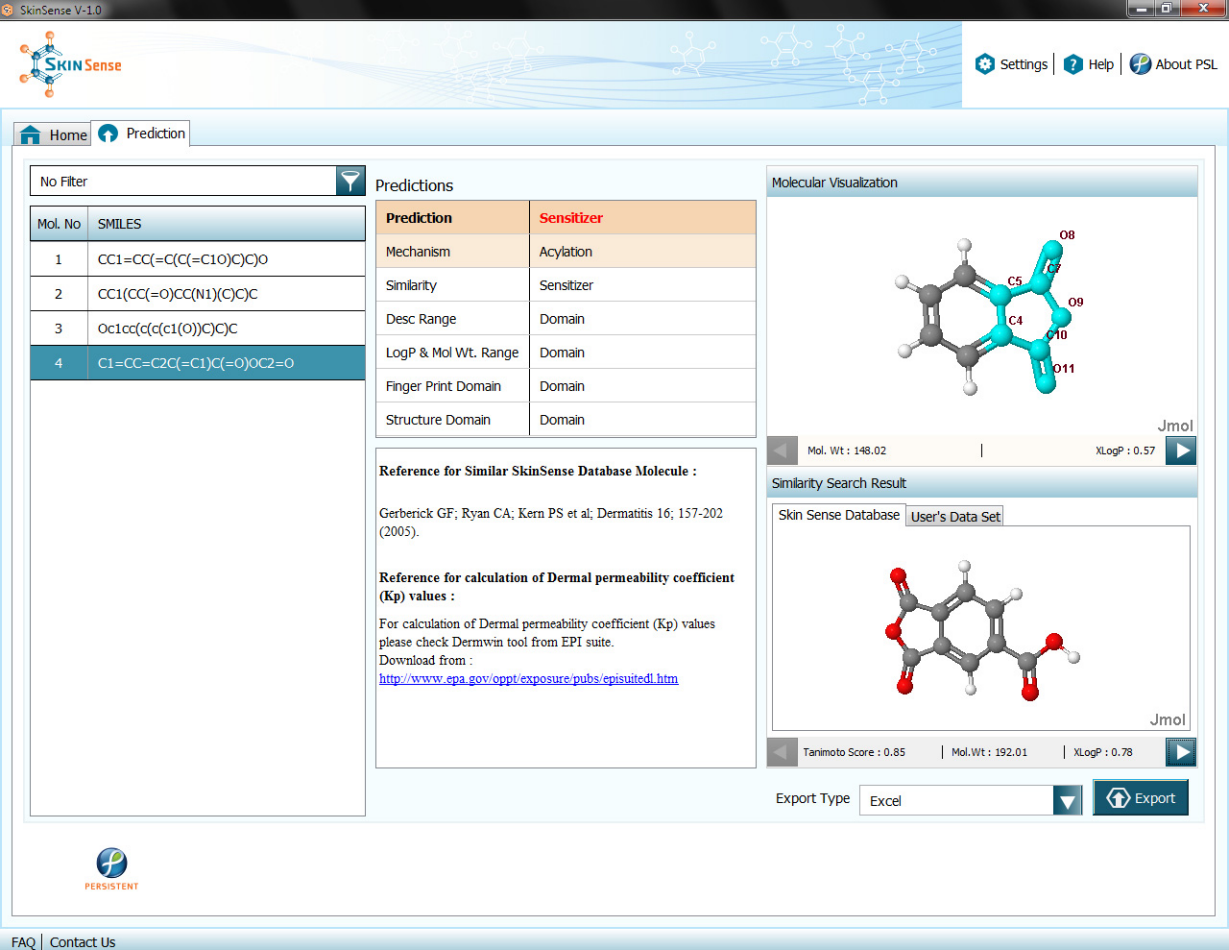
SkinSense–Result Screen. Table on the left shows SMILES of input molecules; ‘Predictions’ section shows prediction result for the selected molecule along with predicted reaction mechanism and domain information; ‘Molecular Visualization’ depicts the structure of selected molecule, along with skin protein reactive sub-structure(s) (if any) highlighted in cyan; ‘Similarity Search Result’ shows parent set molecules found similar to selected input molecule along with details such as Tanimoto coefficient; ‘Export Type’ offers various options to export SkinSense result.

SkinSense is intended to serve as a primary screening tool for dermatology and cosmetic research, which enables prediction of skin sensitization risk of molecules of interest. Our tool also provides mechanistic details such as skin protein reaction mechanisms and highlights reactive groups of molecules (see Fig 6). This would assist in decision making as well as refinement of the molecules early on in the discovery process, and thus, save time and cost.

SkinSense allows import of test molecules, and classifies them into sensitizers and non-sensitizers. It is important to note that SkinSense also predicts reaction mechanisms of test molecules and indicates the reactive group(s) responsible for reaction with skin proteins. This facilitates user to gain insight into the mechanistic details of test molecules, which in turn, allows the mapping to skin sensitization AOP.

The software is currently available free of charge at: http://eskin.persistent.co.in/deskDownloader/skinsense/download-installer, and will soon be released as an open source tool for the scientific community to facilitate further enhancements. Such enhancements may include, for example, flexibility to incorporate new experimental data (such as peptide binding of molecules) in our existing prediction workflows.

## Conclusions

Our integrated computational solution for predicting skin sensitization combined knowledge from known molecules and reaction mechanisms involved in sensitization, with computational methods and heuristics to develop and refine the workflows. This helped us achieve the improved prediction performance (i.e. accuracy = 75.32%, CCR = 74.36%, sensitivity = 70.00% and specificity = 78.72%) for skin sensitization potential of molecules as compared to existing tools. We believe this advancement would benefit the computational screening of molecules, and would be invaluable in the recent initiative of reducing animal usage in cosmetic and pharmaceutical research. Furthermore, the integrative framework outlined in this study may be replicated for predicting other important therapeutically important endpoints.

## Supporting Information

S1 File Supplementary file contains following tables: **Table A.** Descriptors & Fingerprints list.; **Table B:** Descriptors & Fingerprints values for model-1.; **Table C**: Descriptors & Fingerprints values for model-2.; **Table D:** Descriptors & Fingerprints values for model-3.; **Table E**: Descriptors & Fingerprints values for model-4.; **Table F:** Assigned values for machine learning weights.; **Table G:** Knowledge-based optimization.; **Table H:** Prediction accuracy of model variants.; **Table I:** Y-randomization for QSAR model variants incorporated in PW-1 or PW-2; **Table J:** Prediction of molecules of challenge sets by prediction workflow-1 (PW-1); **Table K:** Prediction of molecules of challenge sets by prediction workflow-2 (PW-2); **Table L:** Predicted reactive group(s) and skin-reaction mechanism(s) for molecules of challenge sets-1 and 2.; **Table M:** Prediction performance of VEGA v1.08 (with high reliability scores) with respect to challenge set-1.; **Table N:** Prediction performance of VEGA v1.08 (with high and medium reliability scores) with respect to challenge set-1.; **Table O:** Prediction performance of VEGA v1.08 (with high reliability scores) with respect to challenge set-2.; **Table P:** Prediction performance of VEGA v1.08 (with high and medium reliability scores) with respect to challenge set-2.; **Table Q:** Derivation and comparison of prediction performance of our prediction workflows with existing tools.
(ZIP)
